# Converting monoclonal antibody-based immunotherapies from passive to active: bringing immune complexes into play

**DOI:** 10.1038/emi.2016.97

**Published:** 2016-08-17

**Authors:** Jennifer Lambour, Mar Naranjo-Gomez, Marc Piechaczyk, Mireia Pelegrin

**Affiliations:** 1 ‘Equipe Labellisée par la Ligue Contre le Cancer' - Institut de Génétique Moléculaire de Montpellier, UMR 5535, CNRS, 34293 Montpellier Cedex 5, France; Université de Montpellier, 34090 Montpellier, France

**Keywords:** antiviral therapy, DC, Fc receptors, immunotherapy, immune complexes, monoclonal antibodies, vaccine-like effects, vaccine strategies

## Abstract

Monoclonal antibodies (mAbs), which currently constitute the main class of biotherapeutics, are now recognized as major medical tools that are increasingly being considered to fight severe viral infections. Indeed, the number of antiviral mAbs developed in recent years has grown exponentially. Although their direct effects on viral blunting have been studied in detail, their potential immunomodulatory actions have been overlooked until recently. The ability of antiviral mAbs to modulate antiviral immune responses in infected organisms has recently been revealed. More specifically, upon recognition of their cognate antigens, mAbs form immune complexes (ICs) that can be recognized by the Fc receptors expressed on different immune cells of infected individuals. This binding may be followed by the modulation of the host immune responses. Harnessing this immunomodulatory property may facilitate improvements in the therapeutic potential of antiviral mAbs. This review focuses on the role of ICs formed with different viral determinants and mAbs in the induction of antiviral immune responses in the context of both passive immunotherapies and vaccination strategies. Potential deleterious effects of ICs on the host immune response are also discussed.

## THERAPEUTIC POTENTIAL OF ANTIVIRAL MONOCLONAL ANTIBODIES

Monoclonal antibodies (mAbs) have gained an important place in the therapeutic arsenal against severe human diseases. More than 50 mAbs have been approved or are under review for human use, and several hundred are currently being tested in the clinic,^1, 2^ most of them to treat patients suffering from a variety of cancers or inflammatory diseases.

Concerning antiviral mAbs, only one, directed against respiratory syncytial virus (RSV), has been approved for the prophylactic treatment of pediatric infections. However, employing mAbs as antiviral drugs is under consideration for the treatment of several chronic and acute severe viral infections, especially to address the public health emergencies such as the recent Ebola virus and Middle East respiratory syndrome coronavirus outbreaks.^3, 4, 5, 6, 7^ Illustrating this trend, the number of antiviral mAbs developed and tested in preclinical and clinical trials has grown exponentially in the past 10 years and includes mAbs directed against life-threatening agents, such as human immunodeficiency virus (HIV), hepatitis B virus (HBV), hepatitis C virus (HCV), influenza virus, dengue virus, Ebola virus and severe acute respiratory syndrome virus coronavirus, among others.^8, 9, 10, 11, 12, 13, 14, 15, 16, 17, 18, 19, 20, 21, 22^ Importantly, recent clinical data have also demonstrated the efficacy of anti-HIV mAbs in controlling viremia, when administered to HIV-infected patients, lending strong support to the idea that mAbs could broaden the therapeutic arsenal against severe viral infections.^23, 24^ Their use as antiviral agents is all the more likely to be considered given that multiple biological activities may account for their therapeutic effects.

**Figure 1:**
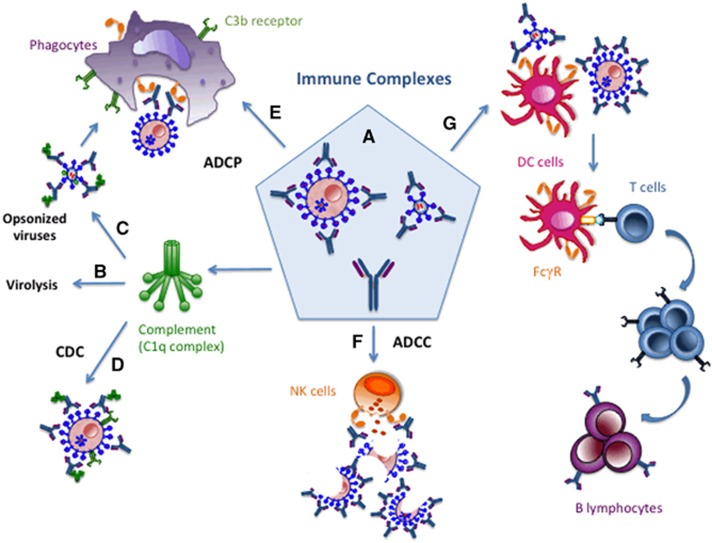
Multiple Fc-mediated activities of antiviral mAbs (monoclonal antibodies). Antiviral mAbs can opsonize viruses, as well as infected cells in situations when viral antigens are also expressed on their surface (**A**). This can lead to viral clearance through various immune-mediated mechanisms. The Fc domain allows the binding of complement to free virions, inducing direct virolysis (**B**). Fcγ- and complement receptors can recognize opsonized virions, leading to their phagocytosis by cells of the innate immune system (**C**). Infected cells can also be eliminated by complement-dependent cytotoxicity (CDC), antibody-dependent cellular phagocytosis (ADCP) and/or antibody-dependent cell-mediated cytotoxicity (ADCC) mediated by innate immunity effector cells expressing FcγRs (**D**–**F**). Immune complexes (ICs) made with mAbs and different viral determinants (virions or infected cells) can be recognized by FcγRs expressed on antigen-presenting cells such as dendritic cells (DCs) (**G**). IC recognition by DCs subsequently leads to enhanced antigen uptake and presentation, allowing the induction of stronger antiviral immune responses.

Although a few mAbs have been developed to inhibit the recognition of viral receptors or co-receptors on the surface of target cells, most antiviral mAbs have been selected for their ability to neutralize virions through the binding of their antigen-binding (Fab) fragment to viral surface antigens essential for entry into host cells. However, the biological activity of antibodies is also mediated by the fragment crystallizable region (Fc) moiety. Thus, it is interesting to note that most antiviral mAbs in use are immunoglobulin (Ig)-Gs displaying a variety of effector functions, including binding to both complement and Fcγ receptors (FcγRs). Different types of FcγRs are expressed in a regulated manner by many cells of the immune system, including B cells, natural killer cells, dendritic cells (DCs), monocytes/macrophages, granulocytes and mast cells, and their engagement by the Fc antibody moiety is essential for regulating the antibody effector functions.^25, 26^ Upon recognition of their target antigens, antiviral mAbs can facilitate virus elimination via two types of complement-mediated mechanisms: (i) inactivation of viral particles and/or phagocytosis of opsonized virus mediated by cells of the innate immune system (Figure 1) and (ii) opsonization and subsequent lysis of infected cells, when viral antigens are also expressed on the cell surface (for example, envelope (Env) glycoprotein of lentiviruses such as HIV) via complement-dependent cytotoxicity. In addition to complement-mediated actions, recognition of FcγRs can entail antibody-dependent cellular phagocytosis and antibody-dependent cell-mediated cytotoxicity (Figure 1).^20, 27, 28, 29, 30^ Finally, antiviral mAbs also have a role in viral blunting by inhibiting cell-to-cell viral transmission.^31^

In addition to controlling the viral propagation by these mechanisms, the opsonization of viral particles and/or infected cells by therapeutic antiviral mAbs of the IgG type leads to the formation of immune complexes (ICs) recognizable by the FcγRs expressed on antigen-presenting cells (APCs) such as DCs. This can potentially affect the endogenous antiviral adaptive immune response of passive immunotherapy-treated individuals. Despite the fact that the immunoregulatory functions of antibodies (as well as ICs) have been known for a long time, and have been reported in different experimental settings and physiopathological situations,^25, 32, 33, 34, 35, 36, 37, 38^ the immunomodulatory role of mAbs with clinical potential as antiviral drugs has only recently been considered.

This review mainly focuses on the induction of antiviral immune responses by ICs in both passive immunotherapies and vaccination strategies. The potential deleterious effects of antiviral antibodies on the host immune dysfunction and/or viral propagation are also discussed.

## ANTIVIRAL MABS CAN ENHANCE THE HOST ANTIVIRAL IMMUNE RESPONSE IN AN FC-DEPENDENT MANNER

Only recently have studies addressed whether and how passive immunotherapies utilizing antiviral mAbs are able to enhance the antiviral immunity in infected individuals. This is largely due to the limited availability of suitable immunocompetent animal models of viral infection that allow in-depth investigations of the endogenous immune response. The concept that passive immunotherapies utilizing antiviral mAbs can induce long-term protective immunity has recently been established using an immunocompetent mouse model, consisting of short immunotherapies given to young animals infected with the FrCasE murine leukemia virus. The induction of such ‘vaccine-like' effects by antiviral mAbs, as well as some of the mechanisms involved, are reviewed in detail elsewhere.^39^ In brief, the inoculation of mouse pups with FrCasE is fatal because the antiviral immune response is too weak to control the viral propagation. In contrast, treatment with a neutralizing mAb for several days shortly after infection blunts viral propagation and induces a lifelong protective antiviral immunity composed of both a highly neutralizing humoral response and a cytotoxic CD8^+^ T-cell response.^40, 41, 42, 43, 44, 45^ This induction of protective immunity strictly depends on the Fc fragment of the neutralizing mAbs.^43, 44^ Moreover, the formation of ICs composed of the administered mAbs and infected cells rather than virions is crucial for the enhanced antiviral immune response.^43^ Such ICs are recognized by the FcγRs expressed by DCs, which facilitate ICs internalization and lead to stronger activation and more efficient antigen presentation by DCs, eventually leading to stronger cytotoxic T-lymphocyte (CTL) responses. An Fc-mediated effect that occurs concurrently is the inhibition of regulatory T-cell (Treg) expansion. This depends on the mAb effector functions^45^ and occurs rapidly. Moreover, it is necessary for the development of the protective humoral and cellular responses, as Treg-mediated immunosuppression is observed in all cases of chronic viral infections, where it dampens antiviral immune responses, thereby permitting the establishment of chronicity. Finally, breastfeeding and placental transfer of maternal anti-FrCasE Igs induced by mAb immunotherapy not only led to the viral propagation blunting in infected pups, but also to the induction of long-lasting protective humoral immunity in these animals.^42^ This is a particularly interesting observation when one considers that the FrCasE model is reminiscent of perinatal infection by HIV, including breastfeeding-mediated mother-to-child virus transmission.

Other evidence for the induction of ‘vaccine-like' effects by antiviral mAbs comes from studies in several preclinical models of human viral infections and from HIV-infected patients.

In a mouse model of RSV infection, the administration of a neutralizing mAb directed against the virus attachment protein G induced a shift in the adaptive immune response from Th2- to Th1-type, leading to sustained and enhanced humoral and CD8^+^ T-cell responses.^46^ However, this effect was not Fc-dependent, but rather due to the ability of the therapeutic mAb to counteract the intrinsic immunosuppressive activity of the RSV G protein.

mAb-driven enhancement of the humoral response has also been reported in two preclinical models of henipavirus infection in African green monkeys.^47, 48^ Recovery from both Hendra and Nipah virus-induced disease correlated with the development of host antibody responses consequent to the administration of the highly neutralizing 102.4 mAb. This Hendra and Nipah virus cross-reactive mAb is currently being considered for human use.

Finally, anti-HIV antibodies can modulate immune responses in infected organisms. Such effects were initially reported in several non-human primate models of HIV infection and then observed in infected humans. Macaques were infected with different strains of simian immunodeficiency virus (SIV) or simian HIV (SHIV, a chimeric virus in which HIV Env substitutes for that of SIV and allows for the assessment of the antiviral effects of anti-HIV antibodies) following different protocols. These experiments showed that the administration of highly neutralizing antibodies (either mAbs or polyclonal Igs) enhanced both the humoral and cellular antiviral immune responses of treated animals.^8, 49, 50, 51^ Interestingly, recent clinical data describe the elicitation of host humoral responses in viremic subjects upon single injection of the potent 3BNC117 anti-HIV mAb.^52^ However, the mechanisms leading to the stimulation of antiviral immune responses in these preclinical models of HIV infection or in infected patients remain uncharacterized. Moreover, it is unknown whether these antiviral responses have genuine protective vaccine-like effects. In any case, these important observations open new avenues for the improvement of mAb-based antiviral HIV therapies. Moreover, as the *in vivo* activity of anti-HIV-1 bNAbs, including viral load control, was recently shown to crucially depend on Fc effector functions,^53, 54^ an important issue is identifying that Fc–FcγRs interactions are involved in the induction of vaccine-like effects by antiviral mAbs.

## ICS ENHANCE DC ACTIVATION AND INDUCE STRONGER ANTIVIRAL T-CELL RESPONSES: EVIDENCE FROM *IN VITRO* STUDIES

To understand the mechanisms underlying the enhancement of antiviral responses by ICs, several *in vitro* studies have addressed whether antibody-mediated viral uptake by DCs could lead to stronger activation of these cells and the development of stronger virus-specific CD4^+^ and CD8^+^ T-cell responses in an Fc-dependent manner. Such an increase in the cellular immune response has been reported in different infectious settings using ICs made with different types of antigens, including recombinant viral proteins and whole virions, as well as infected cells (Table 1).

Concerning ICs made with viral proteins, several reports have shown that ICs made up of anti-HBV mAbs and the hepatitis B surface antigen (HBsAg) can affect DC function and enhance T-cell responses. HBsAg/anti-HBV ICs significantly increased the uptake of the immunocomplexed HBsAg antigen, and augmented the *in vitro* proliferation of virus-specific T cells and their production of interferon (IFN)-γ.^55^ Moreover, DCs from HBV-infected patients incubated with HBsAg/anti-HBV ICs showed higher expression of major histocompatibility complex (MHC)-II molecules and higher production of interleukin (IL)-12. IC-loaded DCs also enhanced production of IL-2 and IFN-γ by co-cultured T cells.^56^ Interestingly, the therapeutic efficacy of HBsAg/anti-HBV ICs has been tested in clinical trials (see below) in HBV-infected patients with encouraging results.^60, 61, 62^ More recently, in experiments aimed at visualizing immunopotentialization by HBsAg/anti-HBV ICs (see below), live-cell imaging revealed that ICs were internalized via the FcγRs of APCs and were subsequently transported through early and late endosomes into lysosomes, where they co-localized with MHC-I and MHC-II molecules.^63^ Consistent with the latter observation, the administration of DCs loaded with HBsAg/anti-HBV ICs to mice increased the number of IFN-γ- and tumor necrosis factor-α-producing CD8^+^ and CD4^+^ T cells. Similarly, in an SIV infection setting, the incubation of APCs with ICs made with a recombinant full-length Gag p55 protein and an anti-p55 IgG increased SIV capsid cross-presentation. Capsid cross-presentation was dependent on FcγR-mediated uptake of the immunocomplexed SIV capsid protein, and required its proteasomal and endosomal degradation to generate stronger Gag-specific CD8^+^ T-cell responses.^57^ From a mechanistic standpoint, these studies indicate that antiviral antibodies might enhance the priming and expansion of virus-specific CD4^+^ and CD8^+^ T cells by both promoting the secretion of key cytokines and facilitating the uptake and cross-presentation of viral Ags by FcγR-expressing DCs.

**Figure 2:**
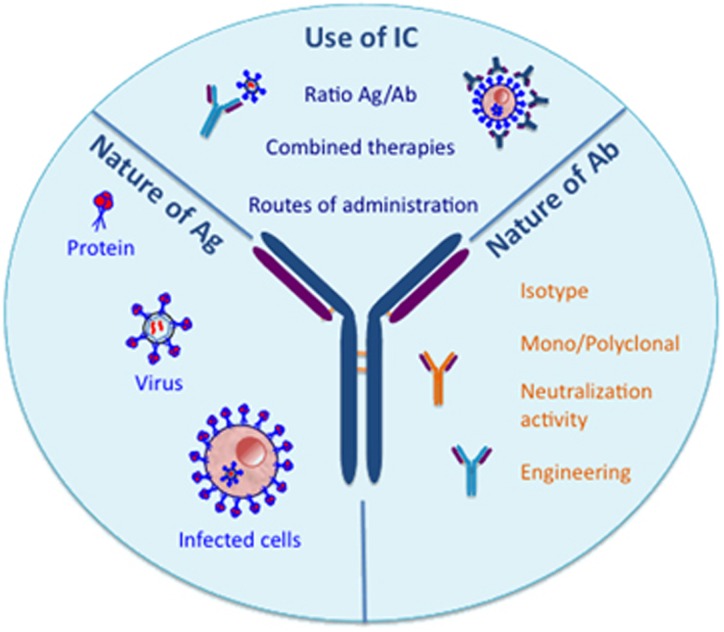
Parameters to consider for achieving optimal IC-mediated modulation of antiviral immune responses. The optimization of vaccine-like-effect-inducing protocols will require the consideration of several parameters such as the nature of the antigen (that is, purified viral proteins, whole virions and infected cells) and the antibody (that is, monoclonal vs polyclonal, nature of the isotype, engineered Fc domain with improved effector functions and so on) used to form the immunogenic ICs, as differences in these parameters might impact immunological outcomes. In addition, whether the optimized ICs are used alone or in combination with immunostimulatory molecules might also be of paramount importance. Several other parameters, including the IC dosage, the route of administration, the choice of adjuvant and the immunological status of patients, will also have to be considered.

Immune-complexed whole virions have also been shown to affect the functional activation of DCs. The stimulation of DCs with ICs composed of SIV virions and highly neutralizing SIV-hyperimmune sera (SVIG) led to the increased virus-specific CD4^+^ T-cell responses in an Fc-dependent manner.^58^ In contrast, DCs stimulated with ICs made of HIV-1 and a polyclonal IgG pool from HIV-infected subjects showed only weak HIV-specific CTL-stimulating activity. This suggested that opsonization of HIV-1 by IgGs might be associated with decreased CTL-stimulatory DC activity.^59^ However, not all IgG isotypes display equivalent effector functions. Therefore, the undefined nature of the antibodies (both in terms of predominant isotypes and neutralization potential) used to form the HIV-ICs in these experiments might explain these observations. Whether HIV-ICs made with highly neutralizing anti-HIV mAbs of a specific IgG isotype might induce stronger CD8^+^ T-cell responses is an important issue deserving further investigation (Figure 2). Moreover, the nature of the viral determinant present in ICs might also be crucial in the stimulation of antiviral responses. Interestingly, as mentioned above, in the mouse FrCasE infection model, ICs made up of a neutralizing mAb and infected cells, but not those made with virions, efficiently induce strong Gag-specific CD8^+^ T-cell responses with high cytotoxic activity.^43^ This observation shows that the viral and cellular ICs can trigger different immunologic outcomes. In the case of FrCasE, this is explained by the fact the FrCasE-GagL CTL immunodominant epitope is, at best, poorly incorporated into virions. Taken together, these data indicate that the uptake of cellular ICs might allow the presentation of a broader viral antigenic repertoire, leading to stronger effects on immunity (Figure 2).

## MODULATION OF ANTIVIRAL IMMUNE RESPONSES BY ICS FORMED WITH ENDOGENOUS ANTIBODIES DURING THE COURSE OF VIRAL INFECTIONS

ICs formed with endogenous antibodies generated in virally infected mice have been shown to influence antiviral cellular immune responses in several models (Table 2). Notably, the highly neutralizing humoral response generated against the FrCasE retrovirus in mAb-treated-infected mice was demonstrated to limit the viral propagation and to enhance memory cellular responses long after the disappearance of the therapeutic mAb (which occurs within two weeks post administration, reflecting the natural IgG lifespan *in vivo*). IC-mediated activation of DCs upon binding to FcγR was key for this effect.^43^ Similarly, in an influenza virus infection model, ICs formed with endogenous antiviral antibodies promoted more sustained antigen presentation by DCs, resulting in stronger CD8^+^ T-cell proliferation.^64^ Interestingly, such prolonged antigen presentation by DCs was dependent on virus-specific, isotype-switched antibodies that facilitated the capture and cross-presentation of viral antigens by FcγR-expressing DCs. In addition, serum antibodies can affect the virus-specific CD4^+^/CD8^+^ T-cell balance in an Fc-dependent manner during RSV infection.^65^ An enhanced ratio of RSV-neutralizing to -non-neutralizing antibodies profoundly enhanced the CD4^+^ T-cell response. In a murine lymphocytic choriomeningitis virus (LCMV) infection model, endogenous virus-specific antibodies could stimulate innate immunity and thereby positively affect both the induction and the maintenance of the virus-specific CD8^+^ T-cell response. Notably, anti-LCMV antibodies limited viral replication in peripheral organs, but allowed replication of the virus in the marginal zone of the spleen, promoting CD8^+^ T-cell priming.^66^ Interestingly, anti-LCMV antibodies were also reported to be essential for long-term maintenance of the memory CTL response in infected mice.^67, 68^

These observations, together with the *in vitro* studies described above, demonstrate that virus-specific antibodies can promote the acquisition, processing and presentation of antigens that are subsequently instrumental for priming T-cell responses and programming functional CD8^+^ memory in an Fc-dependent manner. They strengthen the concept that antiviral antibodies can regulate the quality and function of antiviral T-cell responses through the formation of ICs. Moreover, they also provide a rationale for developing novel IC-based therapeutic vaccination strategies.

## ENHANCEMENT OF ANTIVIRAL IMMUNE RESPONSES IN IC-BASED VACCINATION STRATEGIES

In 1961, Terres and Wolins^69^ demonstrated the ability of ICs to induce higher antibody titers than antigens alone. Since then, the immunogenic potential of ICs, alone or in combination with different types of adjuvants, has been tested in several viral infection systems, including animal models of human infections, for example, those involving HBV, HIV, RSV or flaviviruses. The immunostimulatory are principally attributed to the ability of Fc antibody fragments to recruit the host immune system. However, evidence also implicates Fab fragments in modulation of the antiviral immune response, although the outcomes are less documented and were proposed to occur via alterations in antigen conformation and/or in the exposure of specific epitopes.

We describe the enhancement in antiviral immune responses observed in IC-based vaccination experiments below (Table 3).

### HBV

ICs have been tested as vaccines to augment protective immune responses in different animal models of HBV infection. In ducks, ICs made with duck HBsAg and rabbit anti-duck HBsAg (DHBsAg/anti-DHB) were used as immunogens in the form of solid matrix–antibody–antigen complexes (SMAA). Such SMAAs contained killed *Staphylococcus aureus* as a solid matrix and mAb-opsonized viruses.^79^ They were initially shown to induce both humoral and CTL responses against the paramyxovirus simian virus 5 in immunized mice.^80^ Immunization of HBV-infected ducks with SMAA-based DHBsAg/anti-DHB ICs led to the viral clearance in 60% of infected ducks. Notably, the administration of DHBsAg/anti-DHB ICs lacking *Staphylococcus aureus* showed decreased immunization efficiency, suggesting that the bacteria-based solid matrix functions as an adjuvant.^70^

ICs have also been tested as a therapeutic vaccine against HBV infection in mice^63, 71, 72^ and woodchucks.^73^ BALB/c mice immunized with HBsAg/anti-HBV ICs produced the increased levels of virus-specific antibodies.^71^ Moreover, administering HBsAg/anti-HBV ICs to BALB/c mice via intranasal inhalation induced both mucosal and systemic Th1-polarized immune responses, when administered with adjuvants such as cholera toxin or oligodeoxynucleotides containing immunostimulatory CpG motifs (CpG). This was not observed using HBsAg alone.^74^ In addition, the administration of HBsAg/anti-HBV ICs to HBsAg-positive transgenic mice decreased the serum HBsAg levels and induced stronger CTL responses than HBsAg alone.^72^ Notably, the co-administration of ICs and a plasmid coding for HBsAg increased the antiviral immune response induced by ICs, indicating the adjuvant effect of DNA in this setting. A similar effect was also reported in a woodchuck model of HBV infection: immunization of woodchuck hepatitis virus (WHV)-infected animals with WHV surface antigen/anti-WHV antibody ICs combined with a DNA vaccine resulted in a higher reduction of both viral load and antigenemia relative to ICs alone.^73^ Interestingly, the WHV-infected animals were pretreated with lamivudine (a potent HBV antiviral drug able to enhance T-cell responses in chronically HBV-infected patients) before IC/DNA immunization, suggesting that combination strategies should be considered in treating chronic HBV infections (Figure 2).

The enhancement of antiviral immune responses by ICs *in vitro* and in preclinical models of HBV infection paved the way for the development of IC-based therapeutic vaccination strategies against chronic viral hepatitis B infection. A therapeutic vaccine composed of yeast-derived recombinant HBsAg/anti-HBV immunogenic complexes (YICs) has been tested in a series of clinical trials. This vaccine approach was initially shown to be safe and to induce higher titers of HBsAg antibodies, as well as to increase serum IFN-γ and IL-2 levels in a phase I trial.^60^ Importantly, a subpopulation of chronic viral hepatitis B patients showed a decrease in serum HBV viral load and HBsAg levels together with an increase in anti-HBsAg antibody titers in subsequent phase II trials.^61, 62^ From a mechanistic standpoint, recent data showing that the administration of YIC-loaded DCs to mice increased both CD8^+^ and CD4^+^ T-cell responses^63^ suggest that the improved immune responses induced by YICs might account for the antiviral effect observed in a fraction of patients. In an attempt to enhance the immunogenic potential of YIC-based vaccines, a phase III trial tested the therapeutic effect of a higher number of IC doses. Unfortunately, overstimulation with YIC decreased the vaccine efficiency due to host immune fatigue.^81^ This suggests that vaccination protocols must be optimized and must take into account both the nature and the dose of ICs, as well as other parameters such as the route of administration, the type of adjuvant and the immunological status of patients to achieve efficient protective immunization (Figure 2).

### HIV

In 1988, a study immunized healthy volunteers with HIV peptides.^82^ The authors found that compared with free antigen, recall immunization with ICs induced stronger T-cell responses through uncharacterized mechanisms. Other reports also describe alteration of the anti-HIV response by ICs.^75, 76, 77, 83^ In particular, the immunization of immunocompetent mice with ICs containing a recombinant HIV-1 gp120 Env glycoprotein and a mAb (654-D mAb) directed to the CD4-binding site induced a higher virus-neutralizing antibody response relative to free antigen. As described above, humoral responses were further increased upon the co-administration of ICs and monophosphoryl lipid-A/dimethyldioctadecylammonium adjuvants. Notably, the interaction of the anti-CD4-binding site mAb with HIV-1 gp120 induced conformational changes in the latter, leading to the enhanced antigenicity and immunogenicity of neutralizing epitopes localized in the HIV-1 V3 loop.^75^ These observations highlight the ability of anti-HIV-1 antibodies to induce antigenic alterations in specific HIV-1 gp120 epitopes upon IC formation. Interestingly, further improvement in the immunogenicity of the V3 loop was obtained in ICs generated with gp120 mutants lacking site-specific N-linked glycans.^76, 77, 83^ Taken together, these observations suggest that the ability of ICs to stimulate the induction of neutralizing antibodies is dictated by the nature of the antigen, as well as the specificity and affinity of the mAbs utilized. These results also indicate the potential contribution of Fab-mediated activities in the enhancement of antiviral humoral responses by ICs.

### Flavivirus

Tsouchnikas *et al.*^84^ investigated the influence of immunization with ICs on the specificity of antibody responses using the E protein of the tick-borne encephalitis virus as an immunogen. Mice were immunized with a dimeric soluble form of E (sE) alone or in complex with mAbs specific for each of the three domains of E. The antibody response induced by these ICs was compared with that observed after immunization with sE alone. Unexpectedly, immunization with ICs did not change the extent of the overall antibody response in immunized mice. However, substantially different antibody responses were observed between the different ICs. These differences most likely reflected an epitope-shielding phenomenon and antibody-mediated structural changes that led to the dissociation of the sE dimer. Thus, such phenomena can profoundly influence the fine specificity of antibody responses to the same immunogen and must be considered in IC-based vaccination strategies.

### RSV

As mentioned above, serum anti-RSV antibodies can affect virus-specific T-cell responses.^65^ On the basis of this, Kruijsen *et al.*^78^ tested whether ICs made with the commercial RSV-neutralizing mAb palivizumab could influence adaptive immune response priming after intranasal administration. Substantial anti-RSV T-cell priming and B-cell responses were observed in mice receiving RSV-ICs, resulting in predominant Th1-type CD4^+^ T-cell response and IgG2c antibody responses. Importantly, the ICs also primed anti-RSV CD8^+^ T cells. These data have important implications for the prophylaxis and treatment of pediatric RSV infections. Nevertheless, interactions between ICs and neonatal versus adult innate and adaptive immune systems still need to be investigated because mouse studies have revealed potential antibody-induced neonatal autoimmunity in certain settings.^85, 86^

## ICS AND IMMUNE DYSFUNCTION

In the course of viral infections, the formation of ICs composed of viral determinants and the resulting host humoral responses can potentially produce deleterious effects. Persistent ICs are formed in a variety of chronic viral infections and may lead to unregulated and protracted FcγR signaling. This may lead to immune dysfunction instead of stimulating antiviral immune responses. In this regard, the high levels of ICs formed during LCMV infection interfere with FcγR-mediated activities.^87, 88^ These endogenously formed ICs were shown to outcompete the effector functions of exogenously administered therapeutic mAbs, in particular binding to FcγRs expressed by immune cells. Persistent endogenous ICs are also linked to dysfunctional B-cell responses in HIV infection, including the suppression of antiviral IgA responses and impaired production of neutralizing antibodies (reviewed in Moir *et al.*^89^). The composition of ICs might also negatively affect the efficiency of the antiviral immune response. For instance, the composition of ICs has been shown to be dynamic throughout the course of HIV infections due to changes in both antibody specificities and virion levels. Notably, circulating ICs are initially comprised of antibodies that opsonize both infectious and non-infectious virions. This results in a decrease in the availability of antibodies able to blunt viral propagation. This phenomenon probably contributes to the reduced efficiency of the antibodies generated during acute infection.^90^ Changes in circulating ICs have also been reported in HCV infections. The level of circulating ICs is low in acutely infected patients, whereas chronically infected individuals show a high proportion of immunocomplexed HCV, raising the possibility that ICs may have a role in the pathogenesis of HCV, namely liver damage.^91^ Moreover, the formation of ICs with non-neutralizing antibodies may also lead to the antibody-dependent enhancement of viral infection of FcγR-expressing cells. This happens in several viral infections, including those by the dengue virus.^92, 93, 94, 95^ Along this line, the binding of ICs to FcγRs on monocytes/macrophages can paradoxically suppress innate immunity, induce IL-10 production and bias responses from Th1 toward Th2. This in turn leads to the increased infectious outputs by infected cells via intrinsic antibody-dependent enhancement.^94, 96^

Finally, ICs have also been reported to have a role in increasing viral loads in the context of gene transfer-based vaccination strategies. In the STEP HIV-1 vaccine trial, which evaluated a replication-defective adenovirus type 5 vector vaccine, the ICs formed with pre-existing anti-Adv5 antibodies improved the environment for HIV-1 replication in T cells. This may have been due to the IC-driven activation of a DC–T-cell axis that induces the activation of CD4^+^ T cells and leads to a permissive environment for HIV-1 infection. This environment probably explains the increased propagation of HIV-1 infection among adenovirus type 5-seropositive vaccine recipients.^97^

## IMPROVEMENT OF POTENTIAL VACCINE-LIKE EFFECTS OF MAB-BASED IMMUNOTHERAPIES

Several approaches can be considered to enhance the immunomodulatory potential of antiviral mAbs, both alone and in the form of ICs, in particular through combining neutralizing mAbs and IC-based vaccination strategies with other therapies. A first possibility would consist of inhibiting immunosuppressive mechanisms in infected individuals by either depleting the Treg response, as suggested by Nasser *et al.*^45^ or targeting immune checkpoints, the latter strategy having already led to improved immune responses against both viral infections and cancer.^98, 99, 100, 101^ In addition, the combination of antiviral mAbs with different immunostimulatory agents can also be envisaged. Because the primary structure and glycosylation pattern of the Fc fragment are both essential for antibody effector functions due to their impact on the engagement of type I and type II FcR family members,^26, 27^ Fc engineering might also represent another approach, not only to improve direct antiviral effects, but also to induce stronger vaccine-like effects. In this regard, identification of the main FcRs and FcR-mediated mechanisms involved in enhancing the antiviral immune response will be of utmost importance. Taking into account that the various IgG isotypes display different effector functions and interact differently with FcγRs, the careful selection of antiviral mAb subclasses is also crucial for enhancing antiviral immune responses. Finally, as FcγR polymorphisms have already been associated with differences in viral disease progression and the therapeutic efficiency of anticancer mAbs, it will be important to evaluate the extent to which such polymorphisms can affect the vaccine-like effects induced by mAb-based antiviral immunotherapies.

## CONCLUSION

The therapeutic potential of antiviral mAbs is now widely accepted, and their use as antiviral drugs is increasingly under consideration. The diverse biological activities of these mAbs lead to the direct control of viral propagation and the modulation of antiviral immunity. This provides a novel rationale for their use in diverse prophylactic and therapeutic approaches. The improvement in both humoral and cellular responses achieved through the administration of mAbs, either free or in the form of immunogenic ICs, offers new therapeutic options. The challenge now is to improve our understanding of how ICs convert mAb-based immunotherapies from ‘passive' to ‘active' and to exploit the underlying mechanisms. This conversion will be crucial in reaching the goal of using antiviral mAbs to induce long-lasting protective immunity against life-threatening viral infections.

## Figures and Tables

**Table 1:** *In vitro* studies of T-cell responses modulation by IC-activated DC

**Type of viral determinant**	**Antigen**	**Antibody**	**Immune outcome**	**Reference**
Viral proteins	Recombinant HBsAg	Polyclonal Anti-HBSAg (patients sera)	Increased uptake of IC Augmented *in vitro* proliferation of T cells Augmented production of IFN-γ by T cells	^55^
	HBsAg (purified from patients)	Polyclonal anti-HBV (patients sera)	Enhanced IL-2 and IFN-γ production by co-cultured T cells	^56^
	Recombinant SIV Gag-p55	Polyclonal anti-p55 (monkeys sera)	Enhanced cross-presentation Induction of anti-Gag-specific CD8+ T-cell responses	^57^

Virions	SIV virions	Highly neutralizing SIVIG (monkeys sera)	Enhanced virus-specific CD4+ T-cell responses	^58^
	HIV-1	Polyclonal anti-HIV (patients sera)	Weak stimulating capacity of HIV-specific CTL response	^59^

Infected cells	FrCasE-infected cells	Anti-gp70 mAb	Enhanced proliferation of Gag-specific CD8+ T cells	^43^

Abbreviations: cytotoxic T lymphocyte, CTL; immune complex, IC; interferon, IFN; interleukin, IL; hepatitis B surface antigen, HBsAg; HBV, hepatitis B virus; human immunodeficiency virus, HIV; polyclonal immune globulin prepared from hyperimmune SIV infecetd animals, SIVIG.

**Table 2:** Modulation of immune responses by ICs involving antiviral host humoral responses

**Murine models of infection**	**Immune outcome**	**Reference**
FrCasE virus (MLV)	Enhanced memory cellular CD8+ responses	^43^
Influenza	Prolonged Ag presentation by DC; enhanced proliferation of CD8+ T cells	^64^
RSV	Modification of CD4+/CD8+ T-cell balance	^65^
LCMV	Innate immune activation; induction of virus-specific CD8+ T-cell response	^66^

Abbreviations: lymphocytic choriomeningitis virus, LCMV; murine leukemia virus, MLV; respiratory syncytial virus, RSV.

**Table 3:** Vaccine strategies based on immunogenic ICs

**Antigen**	**Antibody**	**Administration**	**Host**	**Adjuvant**	**Immunological/clinical outcome**	**Reference**
Yeast-derived recombinant HBsAg	HBIG (purified from patients)	Intramuscular	Human (clinical trial)		Decreased viral load Higher titers of HBsAg Ab Increased frequency IFN-γ and IL-2 producing T cells	^60, 61, 62^
DHBsAg	Polyclonal Anti-DHBsAg	Intraperitoneal	Ducks	Bacteria-based solid matrix	Clearance of serum DHBsAg	^70^
HBsAg (HBV vaccine, GenHevac B)	mAb Anti-HBV (1B11)	Intravenous and subcutaneous	BALB/c mice	Aluminum hydroxyde	Enhancement of humoral responses	^71^
HBsAg (S, PreS1, Prs2 domain of HBV)	Polyclonal Anti-HBV	Intramuscular	C57BL/6 HBsAg-positive transgenic mice	DNA plasmid coding HBsAg	Decreased Ag serum levels Induction of CTL responses	^72^
WHVsAg	Polyclonal Anti-WHV	Intramuscular	Woodchucks	DNA plasmid coding WHBsAg	Reduction of viral load and antigenemia	^73^
HBsAg (plasma-derived HBV S protein)	Polyclonal Anti-HBV	Intranasal inhalation	BALB/c mice	Cholera toxin or ODN containing CpG	Mucosal and systemic Th1-polarized immune responses	^74^
Recombinant HIV-1 gp 120	Anti-CD4 (654-D mAb)	Intraperitoneal	BALB/c mice	MPL/DDA	Enhancement of humoral responses	^75, 76, 77^
RSV A2 strain	Anti-RSV (palivizumab)	Intranasal	C57BL/6 mice	—	Priming of virus-specific T- and B-cell responses	^78^

Abbreviations: antibody, Ab; cytotoxic T lymphocyte, CTL; dimethyldioctadecylammonium, DDA; duck HBsAg, DHBsAg; hepatitis B immunoglobulin, HBIG; hepatitis B surface antigen, HBsAg; hepatitis B virus, HBV; interferon, IFN; interleukin, IL; monophosphoryl lipid-A, MPL; oligodeoxynucleotide, ODN; respiratory syncytial virus, RSV; woodchuck hepatitis virus, WHV; WHV antigen, WHVAg.
